# Effects of introduced sika deer (Cervus nippon) and population control activity on the distribution of Haemaphysalis ticks in an island environment

**DOI:** 10.1016/j.ijppaw.2020.03.001

**Published:** 2020-03-05

**Authors:** Kandai Doi, Katsunori Nishida, Takuya Kato, Shin-ichi Hayama

**Affiliations:** a Nippon Veterinary and Life Science University, Laboratory of Wildlife Medicine, 1-7-1 Kyonancho, Musashino, Tokyo 1808602, Japan; b Zephyrus Co. Ltd., Japan

**Keywords:** Haemaphysalis tick, Introduced sika deer, *Cervus nippon*, *Haemaphysalis megaspinosa*, Island environment

## Abstract

The effects of introduced mammal species on the ecology of parasites are often under investigated. The sika deeer, *Cervus nippon*, is host species of many hard ticks. We collected 8348 ticks on an island where sika deer were introduced. The most representative species was *Haemaphysalis megaspinosa* (n = 4198; 50.3%), followed by *H. longicornis* (n = 1945; 23.3%), *H. cornigera* (n = 1179; 14.1%), *H. flava* (n = 713; 8.5%), *Ixodes turdus* (n = 289; 3.7%), *I. granulatus* (n = 22; 0.3%), and *H. hystricis* (n = 2; <0.1%) on an island where sika deer were introduced. *H. megaspinosa* and *H. hystricis* have not previously been recorded on the Izu islands. The high abundance of *H. megaspinosa* indicated that the tick species may have been introduced with the sika deer. Furthermore, *H. megaspinosa* larvae were more abundant at collection sites 21–40 days after sika deer were caught by foot snare traps indicate that engorged female of this tick species were forced to drop off in a very limited area near the foot snare trap. This represented a risk for hunters and people associated with wildlife control visiting the area.

## Highlights

• *Haemaphysalis megaspinosa*, *H. longicornis*, *H. cornigera* were three dominant species of the tick fauna.
• First record of *H. megaspinosa* and *H. hystricis.*
• *H. megaspinosa* larvae was the most abundant at 21–40 days after sika deer was captured.

## Graphical abstract

**Figure:**
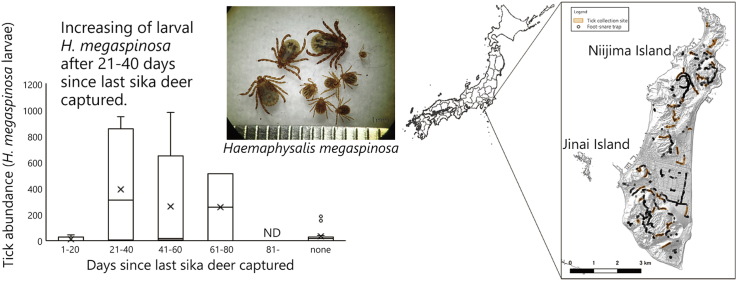

## 1 Introduction

Hard ticks (Ixodidae) are blood-feeding arthropods that infest variety of terrestrial vertebrates, including humans (Randolph, 2004). Blood meals are essential for transstadial development and reproduction but are also an important transmission route for tick-borne disease (TBD) pathogens (de la Fuente et al., 2008; Alexander et al., 2012; Apanaskevich and Oliver, 2014). The emergence of TBDs, such as severe fever with thrombocytopenia syndrome (SFTS) and Japanese spotted fever, in humans and domestic animals has been a major issue in Japan in the last decade (Yamaji et al., 2018; National Institute of Infectious Diseases, 2019). Thus, the ecology, population dynamics, and distribution of ticks are important background information for public health.

Questing ticks are found on vegetation and in leaf litter. Hard ticks have very limited ability to move long distance. However, the movement of hosts, provide to infesting hard ticks an opportunity to spread in other geographical sites. Thus, the tick distribution is often strongly dependent on the distribution of their hosts (Yamauchi et al., 2009; Tsukada et al., 2014).

**Fig. 1:**
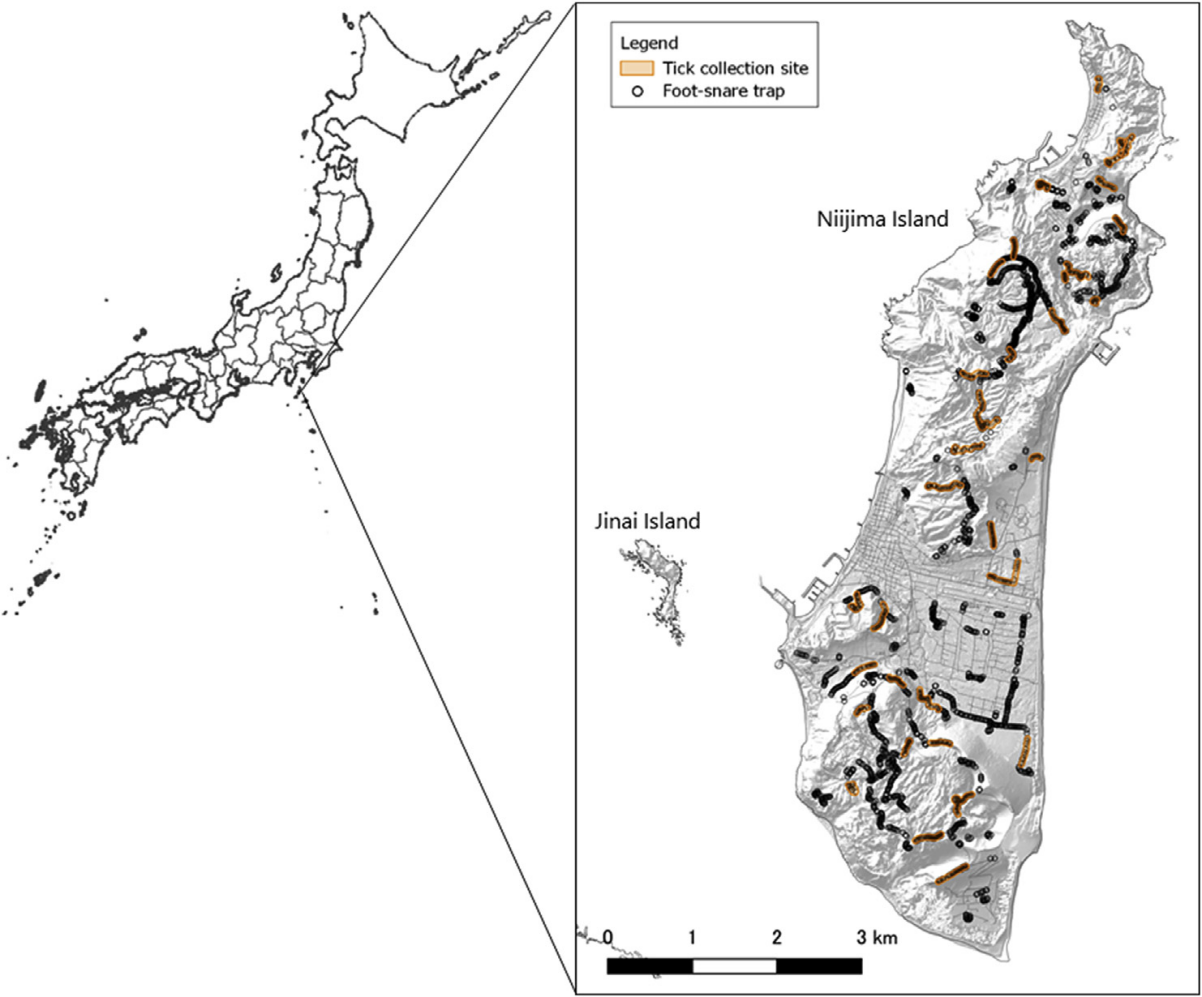
Map of Niijima Island, with locations of foot snare traps (black circle), and tick collection routes for the tick survey conducted June, August, and November of 2018 and February of 2019 (orange area). (For interpretation of the references to colour in this figure legend, the reader is referred to the Web version of this article.)

Cervidae is probably the most common reservoir and host, of TBDs and ticks respectively (Tsukada et al., 2014; Sayler et al., 2016). Sika deer (*Cervus nippon*) is a major ungulate found in Japan (McCullough, 2009). This deer has been introduced in many cities (e.g., Nara; Torii and Tatsuzawa, 2009) for tourism. Niijima Island is one of the Izu Islands, located south of Sagami Bay, Honshu, Japan (Fig. 1). Hasegawa et al. (1994) compiled information on the sika deer population of the island. Seventeen captive sika deer were imported to Jinai Island, an uninhabited island 1.6 km from the west coast of Niijima Island, for tourism in 1969 and 1971 from two zoological parks in Tokyo. However, the sika deer increased in number and started to migrate to Niijima Island in the early 1970s. Between 1974 and 1979, six sika deer were hunted due to crop raiding. In 1993, the village of Niijima implemented a control plan using foot snare traps to eradicate the introduced sika deer population. Since 1993, the village of Niijima set traps in 2250 sites on the island. Every deer captured were euthanized by licensed hunters of the village of Niijima. Carcasses of the captured sika deer were buried in the ground because of the difficulties of transferring carcasses due to the landscape of the island (Ministry of Environment, 2011).

From the late 2000s, local governments started to report an increasing number of human tick infestations (Asahi Shimbun Company, 2010; Ministry of Environment, 2011). Local residents refer to ticks as “sika-dani,” which means “sika deer tick” in Japanese, because they assume that the cases of human infestation were caused by introduced sika deer on the island. However, the tick fauna of the island is not well-studied. Only three scientific surveys have been performed; Nakatsuji (1942) and Asanuma and Kosaka (1955) reported *Ixodes turdus* infestations in birds, and Hasegawa et al. (1994) reported an *I. asanumai* infestation of Okada's five-lined skink (*Plestiodon latiscitatus*) during the 1980s.

This deer on the island were introduced and are the only large mammalian species on that site. The tick fauna of Niijima Island may have been altered by the introduction of this new host (Burridge et al., 2000). In this study, we conducted a survey of the island to investigate the present tick fauna, find tick species brought by sika deer from outside of the island, and determine how the tick distribution was affected by introduced sika deer and the deer population control program.

## 2 Materials and methods

### 2.1 Study site

Niijima Island (34° 22′ 0″ N, 139° 16′ 0″ E), in Tokyo Prefecture, was the study site. The island is about 150 km from Honshu, the main island of Japan, about 3 km east-to-west and 10 km north-to-south (Fig. 1), having two villages, in the north and the center. The northern and southern areas are covered with laurel forest (Yamamoto, 1998).

### 2.2 Tick collection

We conducted tick survey using the flagging method using cotton flannels (75 cm × 50 cm) in June, August, and November of 2018 and February of 2019. Thirty-three tick collection routes were chosen along with the foot snare traps (Fig. 1). The time taken and the number of people involved in flagging for each route were recorded to calculate the tick collection effort (Formula 1). Collected ticks were preserved in 70% ethanol until the morphological identification of species, sex, and developmental stages following Yamaguti et al. (1971) and Fujita and Takada (2007).

### 2.3 Analysis

To illustrate the tick fauna of the island, we determined the dominant species and the relative population density of the tick by estimating the tick abundance (ticks per hour) of each species and developmental stage. The tick abundance was calculated by dividing the number of ticks collected by the tick collection effort (Formula 1 and 2, Dantas-Torres and Otranto, 2013).

We extracted the number of the deer captured and the duration of days between the day of last capture of sika deer to the day of tick collection and the last capture during the previous 90 days for June were determined from the data of the sika deer population control plan. Catch per unit effort (CPUE; Formula 3) was calculated using the number of captured sika deer and trap nights (TN) to estimate the relative usability of sika deer on each collection route.

Routes were categorized into 0–20 days, 21–40 days, 41–60 days, 61–80 days, and ≥81 days according to the sika deer capture records. The survey month with more than 20 routes with the collection of more than one dominant tick was chosen in order to compare the abundance of the larvae of the dominant species with CPUE by using Spearman's rank correlation coefficient (α = 0.05). Furthermore, the larval tick abundance of the dominant species correlated with CPUE was compared with the number of days since the last sika deer capture by generating a box-and-whisker plot to visually evaluate the impact of sika deer capture on reproduction in female ticks that might dropped off from the captured deer. In addition, collection routes were categorized into those with a high sika deer population density (high sika deer) and a low sika deer population density (low sika deer) according to the average CPUE of the collection month to observe differences in the effect on reproduction in drop-off females. To confirm the difference in tick abundance of the dominant tick species between the high sika deer group and the low sika deer group, Welch's *t*-test was used (α = 0.05). All statistical analyses were performed using R 3. 4. 3. (The R Foundation for Statistical Computing, Vienna, Austria).

Formula 1. Tick Collection Effort = time of flagging method × number of individuals involved

Formula 2. Tick Abundance = 60 × number of ticks/tick collection effort

Formula 3. CPUE (sika deer/100 TN) = 100 × Number of sika deer captured since the previous tick survey/TN along the tick collection route

## 3 Results

**Fig. 2:**
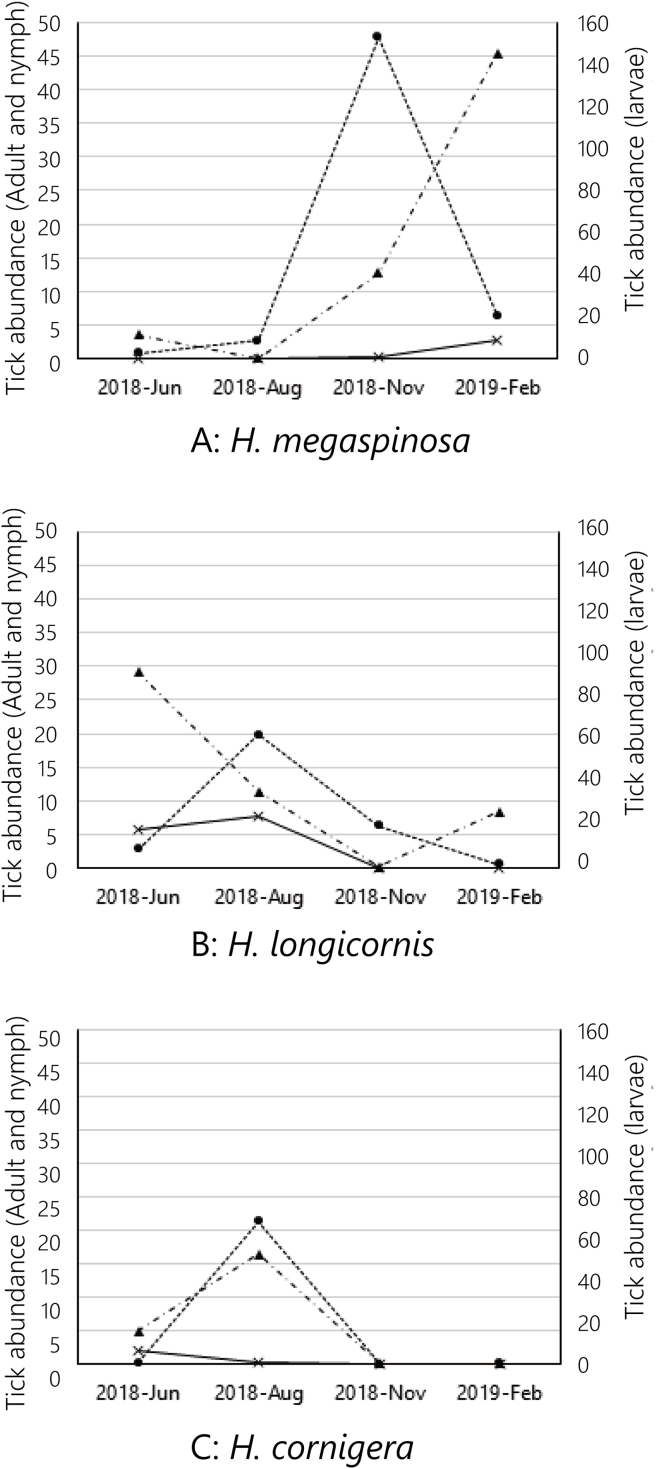
Seasonal changes in tick abundance of the dominant species (A) *H. megaspinosa*, (B) *H. longicornis,* and (C) *H. cornigera* on Niijima Island. (Broken line with black dot: Larvae, Dotted-dashes line with back triangles: Nymphs, Solid line with crosses: Adults).

A total of 8348 ticks were collected and identified. *Haemaphysalis megaspinosa* (n = 4198; 50.3%) was the most commonly collected species, followed by *H. longicornis* (n = 1945; 23.3%), *H. cornigera* (n = 1179; 14.1%), *H. flava* (n = 713; 8.5%), *Ixodes turdus* (n = 289; 3.7%), *I. granulatus* (n = 22; 0.3%), and *H. hystricis* (n = 2; <0.1%) (Table 1). Based on tick abundances, *H. megaspinosa* was the dominant species during the autumn and winter, in November and February (Fig. 2A), and *H. cornigera* and *H. longicornis* were the dominant species during the summer, in June and August (Fig. 2B and C) (see Table 2).

**Table 1:** Tick collection from June 2018 to February 2019. A total of 8348 ticks were collected. *H. megaspinosa* collected mostly in November and February while *H. longicornis* and *H. cornigera* were second and third most species collected in June and August.

		H. megaspinosa				H. cornigera				H. longicornis				H. flava			
		M	F	N	L	M	F	N	L	M	F	N	L	M	F	N	L
2018–Jun	No. of ticks	0	1	26	38	8	8	53	8	0	51	293	96	1	1	7	4
2018–Aug	No. of ticks	0	0	1	113	3	1	288	799	0	96	181	689	0	0	52	28
2018–Nov	No. of ticks	5	2	191	2504	0	0	2	0	0	0	3	408	9	7	153	244
2019–Feb	No. of ticks	32	29	865	391	0	0	1	8	0	0	102	26	15	19	146	27
Total	No. of ticks	37	32	1083	3046	11	9	344	815	0	147	579	1219	25	27	358	303
	Total no. of ticks/Proportion (%)	4198/50.3%				1179/14.1%				1945/23.3%				713/8.5%			

		*H. hystricis*				*I. granulatus*				*I. turdus*							
		M	F	N	L	M	F	N	L	M	F	N	L				
2018–Jun	No. of ticks	0	0	2	0	0	0	0	0	0	0	0	0				
2018–Aug	No. of ticks	0	0	0	0	0	0	0	21	0	0	0	0				
2018–Nov	No. of ticks	0	0	0	0	0	0	0	0	0	0	2	37				
2019–Feb	No. of ticks	0	0	0	0	0	0	1	0	0	2	32	216				
Total	No. of ticks	0	0	2	0	0	0	1	21	0	2	34	253				
	Total no. of ticks/Proportion (%)	2/<0.1%				22/0.3%				289/3.7%

**Table 2:** Mean ± SD of days from the last sika deer capture, CPUE, and tick abundance for *H. megaspinosa* larvae in each survey month.

	Days since last sika deer captured	CPUE	Tick abundance (*H. megaspinosa* larvae)
2018 Jun.	29.25 ± 26.68	0.09 ± 0.14	2.96 ± 9.20
2018 Aug.	63.33 ± 37.62	0.03 ± 0.05	8.79 ± 27.81
2018 Nov.	42.00 ± 27.08	0.09 ± 0.18	152.67 ± 267.5
2019 Feb.	33.33 ± 22.54	0.04 ± 0.10	22.36 ± 48.12

According to records for the sika deer population control plan in the village of Niijima, 57 sika deer captures were recorded along our tick collection routes and the average duration from the last sika deer was 42.5 days (1 ≤ x ≤ 133) from April 2018 to February 2019. The average CPUE values were 8.6 sika deer/100 TN for the previous 90 days for June, 2.6 sika deer/100 TN for June–August, 9.1 sika deer/100 TN for August–November, and 20.1 sika deer/100 TN for November–February.

To determine the relationship between the larval tick abundance of the dominant species and CPUE, we chose seasons and tick species according to Spearman's rank correlation coefficients. *H. megaspinosa* abundance in November was significantly and positively correlated with CPUE (*r* = 0.68, P < 0.05), while the abundance of *H. cornigera* and *H. longicornis* were not significantly correlated with CPUE (*H. cornigera*: *r* = 0.21, P > 0.05; *H. longicornis*: *r* = 0.20, P > 0.05).

**Fig. 3:**
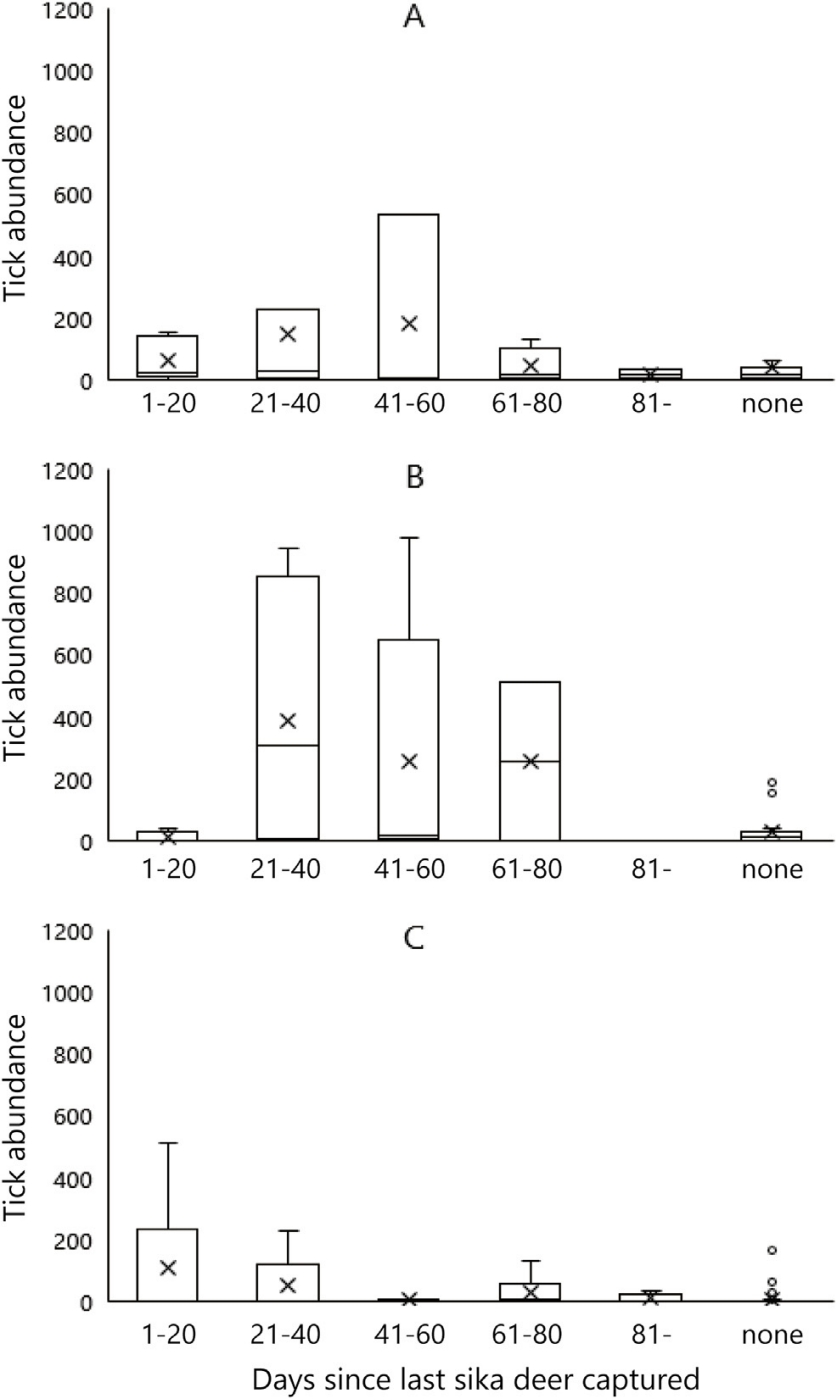
Statistical summary of the abundances of *H. megaspinosa* larvae. (A) Days since the last sika deer was captured in August–November, (B) days since the last sika deer was captured in August–November for the high sika deer group, and (C) days since the last sika deer was captured in August–November for the low sika deer group. (X mark inside box: mean, lower and upper side of the box: first and third quartiles, line inside box: median, lower and upper error lines 10th and 90th percentiles, respectively, circles: data falling outside 10th and 90th percentiles).

Tick abundance for larval *H. megaspinosa* in November reached a peak at 21–40 days after the last sika deer was recorded in August–November (Fig. 3). We divided the sika deer into two groups, the high sika deer group and the low sika deer group according to the average CPUE of the season (CPUE = 9.1 sika deer/100 TN). In a comparison between the high sika deer group (CPUE > 9.1) and the low sika deer group (CPUE < 9.1) in August–November, Welch's *t*-test indicated that there was a significant difference in the abundance of *H. megaspinosa* larvae (t = 3.83, df = 7.39, P < 0.05). Fig. 3-A and B show that the abundance of *H. megaspinosa* larvae peaked 21–40 days after last sika deer was captured, while Fig. 3C shows that the low sika deer density group did not follow the same trend. The trend was stronger for the high sika deer group than for the low sika deer group (Fig. 3A–C).

## 4 Discussion

This study provides the present information of tick fauna of Niijima Island and effects of the deer introduction on *H. megaspinosa* tick abundance. *H. megaspinosa*, *H. longicornis*, *H. cornigera*, *H. flava*, *I. turdus*, *I. granulatus*, and *H. hystricis* were the tick species collected. Out of these 7 species, three dominant tick species, *H. megaspinosa*, *H. cornigera*, and *H. longicornis*, were found. The seasonal abundance of these three species consistent with previous findings (Tsunoda, 2012). For other species, it was difficult to evaluate seasonal abundance owing to the low sample sizes. Compared with previous tick collection studies of Izu Islands (Nakatsuji, 1942; and Asanuma and Kosaka, 1955; Hasegawa et al., 1994), we obtained new records of *H. megaspinosa* and *H. hystricis*. *H. hystricis* had the lowest abundance, that is possible that the species does not yet inhabit Niijima Island. The individuals of *H. hystricis* is possibly transported by avian migration, and animal importation via human transportation which are the possible routes that ticks were found in the geographical region where the individuals have not been belong to (Miyamotoi et al., 2000; Raghavan et al., 2019). *H. megaspinosa* was a dominant species on the island in the autumn and winter, has not previously been reported throughout Izu Islands. This tick species was known that typically prefers large ungulate hosts (Yamauchi et al., 2009; Tsunoda, 2012; Tsukada et al., 2014). It is likely that *H. megaspinosa* was an alien tick species which was introduced to Jinai Island via the sika deer, which subsequently migrated to Niijima Island (Hasegawa et al., 1994).

The positive correlation between larval *H. megaspinosa* abundance and sika deer CPUE was indicated from the comparison between the abundance of *H. megaspinosa* larvae, and the days from sika deer capture by the foot snare traps along the tick collection routes in August–November. In addition, Tsunoda and Fujimagari (1996) measured the duration of oviposition in *H. longicornis* in the laboratory environment and reported that engorged females took 6–8 days to begin oviposition after the blood-meal, with 24–26 days for larva to hatch from eggs. Thus, *H. megaspinosa* larvae, with peak at 21–40 days after sika deer capture which was observed in the result, were probably produced by multiple infested female ticks (Fig. 3-A and B). The ticks detected the death of their host and dropped off around a foot snare trap or the carcass. It is possible that partially engorged female ticks lay fewer eggs than completely engorged females (Ma et al., 2016). However, some proportion of female ticks that dropped off must not be able to complete their blood-meal, and larval ticks may exhibit substantial aggregation around the foot snare trap.

The results of comparison among CPUE, tick abundance, and the days since the last sika deer was captured, indicated evidences of sika deer introduction and sika deer population control plan affected *H. megaspinosa* tick distribution. However, other free-range wildlife such as rodents, feral cat may have been infested by ticks and dispersed to other geographical sites (Hasegawa et al., 1994). Especially, the species in which abundances were not correlated with CPUE may have affected such hosts. Also, abiotic factors, such as humidity and temperature of the sites have affected among survival rate of questing ticks. Therefore, the abundance of sika deer and sika deer population control plan influenced dominant tick species of the island, but other free-ranging wildlife and abiotic factors have also been potentially influencing the tick fauna of the island.

The study focused on the ecological information of the ticks and the deer of the island, however noting the possibilities that these hard tick species found on the island will transmit TBD pathogens are important for basic knowledge for the early detection of possible TBD emergence in the future. The fact that local people have already experienced tick bite on the island (Asahi Shimbun Company, 2010), *I. granulatus* is the vector of *Babesia microti*, which cause human babesiosis (Van Peenen et al., 1977). *H. megaspinosa*, *H. longicornis*, *H. cornigera*, *H. flava*, *H. hystricis*, and *I. turdus* are known as the vector of spotted fever group rickettsia (i.e. Japanese spotted fever, Queensland spotted fever, Rocky mountain spotted fever etc.) (Parola et al., 2013). Izu Islands include Niijima Island, are the area where tsutsugamushi disease, *Orientia tsutsugamushi* infection, is endemic (Miyairi et al., 1978). Symptoms of this tsutsugamushi disease, fever, erythema on skin, and sense of fatigue, are very similar to rickettsiosis (Sando et al., 2018). Both infections are cured by using tetracycline antibiotics (Biggs et al., 2016). Thus, the cases of rickettsiosis which were diagnosed as tsutsugamushi disease may have existed in the past. In addition, these possible neglected cases may have transmitted by the ticks came with introduced sika deer. *H. megaspinosa*, *H. longicornis*, *H. cornigera*, *H. flava*, *H. hystricis* were also known as the vector of SFTS virus. Although the cases of SFTS were only found in western region of Japan, people or pet animals may be going to introduce this virus to the island where multiple vector and reservoir species are present.

The fact that population control plan using the foot snare trap made larval *H. megaspinosa* aggregated where hunters and people associated with wildlife control visit frequently and increase the risk to be exposed by ticks, and tick fauna of the island includes potential vector of variety of TBD pathogens. Thus, for wildlife population control using traps, extra care must be taken to immobilize and leave carcasses in the environment to decrease the risk of tick bites and the emergence of TBDs.

## Declaration of competing interest

We wish to confirm that there are no known conflicts of interest associated with the publication and there has been no significant financial support for this study that could have influenced its out come. We confirm that our manuscript has been read and approved by all named authors and that there are no other persons who satisfied the criteria for authorship but are not listed. We further confirm the order of authors listed in the manuscript has been approved by all of us. We confirm that we have given due consideration to the protection of intellectual property associated with this work and that there are no impediments to the publication, including the timing of publication, with respect to intellectual property. We further confirm any aspect of the work covered in this manuscript that has involved either experimental animals or human patients has been conducted with the ethical approval of all relevant bodies and that such approval are acknowledged within the manuscript. We understand that the Corresponding Author is the sole of contact for Editorial process (including Editorial Manager and direct communications with the office). He is responsible for communicating with the other authors about progress, submissions of revisions and final approval of proofs. We confirm that we have provide a current, correct email address which accessible by the Corresponding Author and which has been configured to accept email from (kandai.doi@gmail.com). Signed by all authors as follow K. Doi, K. Nishida, T. Kato, and S. Hayama (12/26/2019).
